# 

**Published:** 1895-07-20

**Authors:** 


CONTENTS.

**The Misuse of Hospitals;—Consultants and General Practitioners** 263

**Wood Pavement and Disinfectants** 264

**On Modern Progress in Ophthalmic Medicine and Surgery.** By R. B. CARTER, F.R.C.S.—Diseases of the Cornea 265

**Medical Progress and Hospital Clinics—**

Spasmodic Torticollis 269

PROGRESS IN MEDICINE.—Diseases of the Respiratory System (*continued*) 269

PROGRESS IN SURGERY.—Cerebral Surgery (*continued*) 271

PROGRESS IN OPHTHALMOLOGY 273

NEW APPLIANCES AND THINGS MEDICAL 274

**Social Problems.**—Labour and Landlords 266

**Annotations.**—The Scientific Manufacture of Poets—Clinical Research—Medical Politicians 267

**Notes and News** 268

**The Institutional Workshop—**

THE AMBULANCE IN PEACE AND WAR 275

HOSPITAL ADMINISTRATION 275

THE STORY OF THE INSANE FROM YEAR TO YEAR 276

PRACTICAL DEPARTMENTS 277

AROUND THE HOSPITALS 277

CONSTRUCTION NOTES 278

**Scraps and Gleanings** 278

EXTRA SUPPLEMENT.—THE NURSING MIRROR.

**News from the Nursing World.**—In the Service of the Queen—Our Princess—At the Queen's Hall—Attention—Asylum Attendants and Nurses—Should Children be Admitted?—District Nurses at Plaistow—The Use of a Sheet—A Maid of All Work—District Nursing at Broughty Ferry—Attendants' Wages in Sligo—At the Crystal Palace—Registered Nurses' Society—Matrons and Doctors—A Satisfactory Collection—Short Items ciii

**Appointments—Presentation** cv

**Elementary Anatomy and Surgery for Nurses.** By W. MCADAM ECCLES, M.D., M.S., F.R.C.S. XXVI.—The Special Senses cv

**Our American Letter—Wants and Workers** cvi

**New South Wales.**—An Australian Poorhouse cvii

**Everybody's Opinion—Notes and Queries** cvii

**Royal British Nurses' Association** cviii

**Care of the Sick in Alexandria and Cairo.** IV.—The Jews' Hospital cviii

